# Independent validation of machine performance check for the Halcyon and TrueBeam linacs for daily quality assurance

**DOI:** 10.1002/acm2.12391

**Published:** 2018-07-17

**Authors:** Yuting Li, Tucker Netherton, Paige L. Nitsch, Song Gao, Ann H. Klopp, Peter A. Balter, Laurence E. Court

**Affiliations:** ^1^ The University of Texas Graduate School of Biomedical Sciences at Houston Houston TX USA; ^2^ Department of Radiation Oncology The Ohio State University Wexner Medical Center Columbus OH USA; ^3^ Department of Radiation Physics Division of Radiation Oncology The University of Texas MD Anderson Cancer Center Houston TX USA

**Keywords:** Halcyon, MPC, TrueBeam

## Abstract

**Purpose:**

To evaluate the ability of the machine performance check (MPC) on the Halcyon to detect errors, with comparison with the TrueBeam.

**Methods:**

MPC is an automated set of quality assurance (QA) tests that use a phantom placed on the couch and the linac's imaging system(s) to verify the beam constancy and mechanical performance of the Halcyon and TrueBeam linacs. In order to evaluate the beam constancy tests, we inserted solid water slabs between the beam source and the megavoltage imager to simulate changes in beam output, flatness, and symmetry. The MPC results were compared with measurements, using two‐dimensional array under the same conditions. We then studied the accuracy of MPC geometric tests. The accuracies of the relative gantry offset and couch shift tests were evaluated by intentionally inserting phantom shifts, using a rotating or linear motion stage. The MLC offset and absolute gantry offset tests were assessed by miscalibrating these motions on a Halcyon linac.

**Results:**

For the Halcyon system, the average difference in the measured beam output between the IC Profiler and MPC, after intentional changes, was 1.3 ± 0.5% (for changes ≤5%). For Halcyon, the MPC test failed (i.e., prevented treatment) when the beam symmetry change was over 1.9%. The accuracy of the MLC offset test was within 0.05 mm. The absolute gantry offset test was able to detect an offset as small as 0.02°. The accuracy of the absolute couch shift test was 0.03 mm. The accuracy of relative couch shift test of Halcyon was measured as 0.16 mm.

**Conclusion:**

We intentionally inserted errors to evaluate the ability of the MPC to identify errors in dosimetric and geometric parameters. These results showed that the MPC is sufficiently accurate to be effectively used for daily QA of the Halcyon and TrueBeam treatment devices.

## 1 INTRODUCTION

Daily quality assurance (QA) of medical linear accelerators (linacs) is standard practice in radiotherapy clinics. These QA tests include checks of x‐ray output, energy, and beam profile constancy as well as image‐guided radiation therapy (IGRT) functionality as recommended by the American Association of Physicist in Medicine (AAPM) Task Group (TG) 142,^1^ MPPG5a,^2^ and TG‐179.^3^ In general, several devices are used each morning to perform this QA. Beam measurements are usually done with a dedicated beam check device containing multiple diodes and/or ion chambers. IGRT imaging checks are generally done with an imaging phantom that checks the alignment between treatment and imaging geometries as well as the accuracy of IGRT‐guided couch shifts. This process can be time‐consuming, adding 15 min (or more) to morning QA, and can have a high level of user dependency, especially the imaging tests.

Varian Medical Systems (Palo Alto, CA, USA) recently released the Machine Performance Check (MPC) system, which is a fully integrated self‐check tool for assessing the performance of the linac. MPC uses a vendor‐supplied phantom which is placed on the couch at the H2 position, using either a separate bracket or a bracket that is already attached to the phantom (depending on the system). Images of the phantom are acquired with various combinations of collimator positions, gantry angles, and couch positions through an automated sequence. Beam performance checks include beam output constancy, beam uniformity, and beam center shift. Geometry checks include the radiation isocenter size and a measure of coincidence with MV and kV imaging isocenters, as well as checking collimator and gantry readout accuracy, MLC and jaw positioning, and couch positioning accuracy. The MPC tests are executed as a special mode on the linac. All motions (gantry, collimator, and couch) are automatic, as is the analysis of the images. The MPC results are then presented relative to a baseline for the beam performance checks, and compared to absolute specifications for the geometric tests. Users are not able to modify the MPC results or thresholds but can set beam baselines. MPC is available for both the new Halcyon (Varian Medical Systems) and the existing TrueBeam platforms (version 2.0 and newer). The tests on these two treatment delivery systems are similar, although the vendor‐set thresholds vary between the systems. Other differences include the following:

- The Halycon has a “virtual isocenter” where the patient (phantom) is setup outside of the treatment bore, and then moved to the true isocenter by couch motion. This movement is checked by the MPC for the Halcyon.
- TrueBeam systems include kilovoltage imaging, which was not available on the preclinical Halcyon 1.0 unit tested in this work.
- The Halcyon does not allow couch rotations.
- The MPC is part of a built in safety mechanism on the Halycon. On these, unit beams cannot be run in clinical mode unless the MPC has been run and passed on to that calendar day.
- The TrueBeam uses the isocal phantom, also used for imaging geometry calibrations, with a separate couch mount while the Halycon uses a similar phantom that is permanently attached to a couch mount.

Two groups have previously evaluated the stability and sensitivity of MPC test for the TrueBeam system.^4^, ^5^, ^6^, ^7^ Clivio et al. and Barnes et al. compared the results of MPC beam constancy tests with those of other routine QA tests over 10 days and 5 months, respectively. Barnes et al. also intentionally miscalibrated the machine to test the sensitivity of some of the geometric tests. In the present study, we focused on MPC tests available with the Halcyon system. These are compared with those on the TrueBeam system. Our results provide insight into the reliability of the MPC design and identified potential failure mode of the MPC for both the Halcyon and TrueBeam systems, allowing us to incorporate MPC into our daily QA replacing the legacy daily QA systems.

## 2 METHODS

Here, we summarize each specific MPC test, and then describe how we intentionally inserted errors and evaluated the ability of the MPC to detect these errors. Specific details on the MPC system can be found in the publications by Clivio and Barnes and in the vendor documentation.

To assess the ability of the MPC to detect changes in machine output or beam profile, we simulated errors by fully or partially inserting solid water into the beam path to change the linac's apparent output and symmetry. The MPC results were compared to measurements made with a 2D ion‐chamber array (IC profiler, Sun Nuclear, Melbourne FL). Other groups have previously benchmarked this device for a variety of beam measurement, including profile measurements, symmetry, and flatness measurements, and relative output measurements.^8^, ^9^ For the geometric tests, we used a remotely controlled stepping motor to translate or rotate the MPC phantom during the automated acquisition sequence to simulate inaccurate system motions. These tests are summarized in Table 1. These were performed on a preclinical Halcyon 1.0

**Table 1:** Summary of measurements for MPC evaluation

MPC test	Measurement method	
	TrueBeam	Halcyon
Beam constancy — output change	Attenuate the entire beam by solid water slabs	
Beam constancy — uniformity change	Attenuate half the beam by solid water slabs	
MLC position	NA	Miscalibrate one MLC position
Absolute gantry offset	NA	Miscalibrate gantry position
Relative gantry offset	Rotate the MPC phantom during MPC test	
Absolute couch position	Shift the MPC phantom	
Relative couch shift	Shift the phantom during the MPC test	
Virtual to isocenter shift	NA	Shift the MPC phantom during the MPC test

Note, when it was not possible to simulate errors, using external means (e.g., by shifting the MPC phantom), we miscalibrated the motions for the preclinical Halcyon unit only.

### 2.A Beam constancy tests

#### 2.A.1 MPC tests

For both the TrueBeam and Halcyon linacs used in this study, the output and uniformity baselines were established after annual calibration measurements, following all instructions and advice in the Instructions for the User document provided by the vendor (Varian). The MPC beam constancy checks are performed with the gantry and collimator at 0°, the field size fixed at 18 × 18 cm^2^ with the couch/MPC phantom retracted. A ratio image is calculated between the new image and a previously defined baseline image. The central 13.3 × 13.3 cm^2^ portion of this is then used for beam output and uniformity analysis. The idea of using this inner portion of the image is to reduce the impact of jaw (MLC) positioning on the beam constancy calculations.

Output change is given as the average percentage change in detector response in this central region of the imager. The threshold of output change is ±4% for the Halcyon and ±2% for the TrueBeam. As with all MPC thresholds, these are set by the manufacturer and are not user adjustable.

The uniformity change is defined as the variation between the highest and lowest ratio of pixel values (after high frequency noise filtering) to the baseline (eq. (1), where x and y represent the new image pixel values and the baseline pixel values).

(1) $$\%Uniformity\;Change=100\times \left(\max(Ratio(x,y))-\min(Ratio(x,y))\right)$$

Again this is calculated for the inner region of the image. It represents the worst‐case. The threshold of uniformity change is ±2% for both TrueBeam and Halcyon.

#### 2.A.2 Error‐detection tests

**Figure 1:**
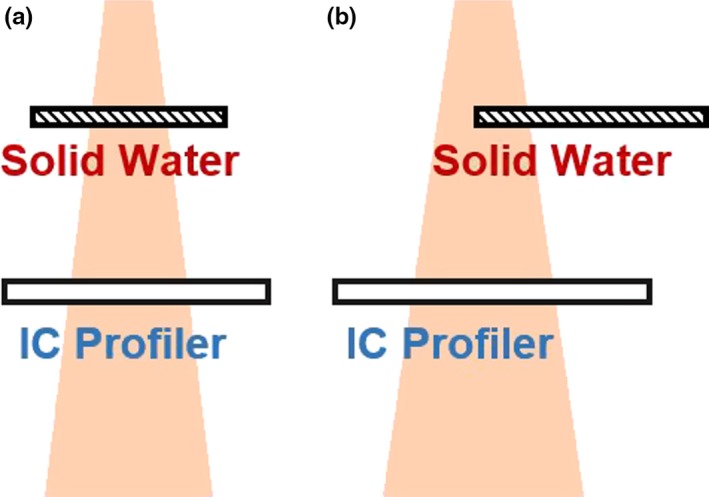
Illustration of measurement setup for (a) output (a) and (b) uniformity tests.

To evaluate the sensitivity of the MPC beam output and uniformity constancy measurements, we inserted solid water slabs between the beam source and the MV imager to introduce changes in beam output or flatness and symmetry of the beam (Fig. 1). The solid water was positioned 40 cm from the isocenter. A 2D‐array (IC Profiler, Sun Nuclear Corporation, FL, USA) was used to verify the MPC measurements of the output, flatness, and symmetry compared to a baseline acquired with no additional material in the beam. For this study, we used the Variance and Local Point Difference calculations in the Profiler control software to describe flatness and symmetry, respectively. Variance describes half the maximum percent variation within 80% of the field size. Local Point Difference is the maximum percentage difference between symmetric points, again within 80% of the field size.

For the beam output tests, solid water slabs were added to uniformly attenuate the entire beam profile. Total thicknesses used were 1, 2, 3, 4, 5, 10, 25, and 26 mm. For beam uniformity tests, water slabs (0, 1, 3,4, 5 mm thicknesses) were used to only block half the beam, creating beams that where neither flat nor symmetric.

### 2.B MLC position test

#### 2.B.1 MPC tests

The MPC MLC positioning test uses an image of a static MLC comb‐shaped pattern. The position of each leaf is determined in these images and compared to the intended position. The MLC position is defined as the distance of the MLC tip from the MLCs center line, where the center line is determined as the line through the center of rotation of the MLC, perpendicular to the MLC direction of movement.

Similar images are also used to check the collimator angle readout and to find the position of the radiation isocenter relative to the collimator rotation axis. The mean and maximum differences between intended and measured positions for all the leaves in each MLC bank are included in the MPC report.

#### 2.B.2 Error‐detection tests

To assess the accuracy of the MPC MLC positioning test for the Halcyon system, we intentionally miscalibrated one MLC position by 0.5, 1.0, 1.5, 2.0 mm, and 5.25 mm. This was achieved by changing the MLC offset — this is a setting not accesible to users, and required Varian engineering support. The MLC position values reported by MPC were compared with these known miscalibrated values. The accuracy of MPC MLC positioning for the TrueBeam has been well studied by Barnes et al., and was not repeated in this work.

### 2.C Couch translation tests

#### 2.C.1 MPC tests

For the TrueBeam system, the MPC measures the couch travel distance between two points with a threshold of ±0.7 mm for lateral and longitudinal translation and ±1.2 mm in the vertical. For the Halcyon system the threshold is ±0.5 mm in all directions. On the Halcyon, the MPC also assesses the translation of the couch from the virtual isocenter outside the bore to the treatment isocenter with a threshold of ±2.0 mm — this large threshold is reasonable as the outer lasers are only used for a rough setup and the manufacturer has required that all patients treated on the machine will get daily IGRT for the final setup. The couch shifts (for shifts inside the bore) are measured based on the fiducials visible in the images. The translation of the couch from the virtual isocenter outside the bore to the treatment isocenter starts at a fixed couch coordinate for which, if the couch coordinates are consistent, the MPC phantom should be aligned with the external lasers. The MPC does not explicitly check this, and the user would have to intervene if this was not the case. The couch is then moved into the bore and the couch positioning at the isocenter is confirmed using images of the phantom.

#### 2.C.2 Couch error detection tests

**Figure 2:**
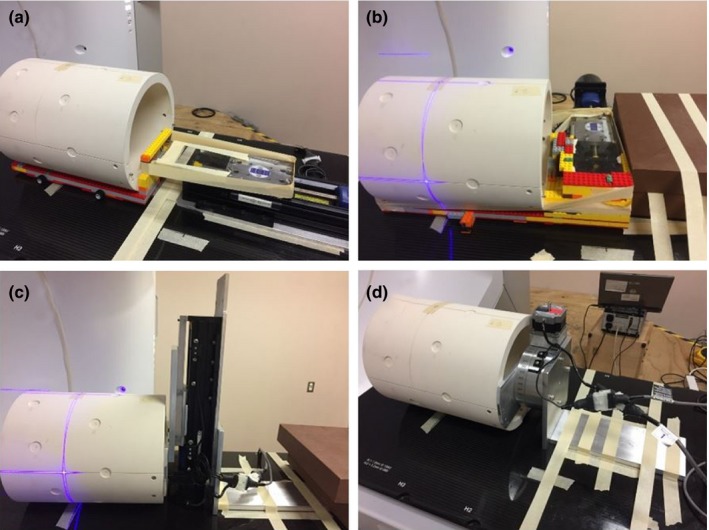
(a–c) Measurement setup for MPC couch translation tests of the Halcyon system. These pictures show the setup for shifting the phantom (a) longitudinally, (b) laterally and (c) vertically. (d) Measurement setup for MPC relative gantry rotation test of Halcyon.

A stepper motor controlled linear stage (BiSlide, Velmex Inc., Bloomfield, NY, USA) was used to shift the MPC phantom during the MPC couch translation tests [Figs. 2(a), 2(b), 2(c)]. The support bracket which is attached to the Halcyon phantom was removed, and the phantom was positioned on a lego stage which was attached to the linear stage. After the first image of the couch position/motion sequence was taken, we shifted the phantom 0.0, 0.3, 0.5, 1.0, 2.5, or 5.0 mm while the couch was being moved to acquire the second image, thus simulating an incorrect couch motion. The stepper motor/linear drive system has a specification accuracy of 0.08 mm. The accuracy was confirmed to be <0.5 mm visually over a 10 cm travel, using a ruler. In order to evaluate the impact of using the stage on the results, the 0 mm shift results were compared with the results when the MPC phantom was setup, using the usual approach (with the bracket at H2). For the TrueBeam, the vertical couch shifts were not examined because the clearance between the linac and the linear motor stand was insufficient when the motor stand was mounted in a vertical orientation.

The movement of the couch to the actual isocenter was evaluated in the same way, except that no images were taken with the phantom at the first position (i.e., at the virtual isocenter outside the bore), so the phantom was shifted prior to initiating the MPC sequence.

### 2.D Gantry rotation tests

#### 2.D.1 MPC tests

The MPC measurement of the absolute gantry position is based on the coincidence of the couch's vertical axis with the central beam axis when the gantry is at 0°. That is, 0° is taken as the situation when a movement along the vertical couch axis does not give any lateral or longitudinal shift of the phantom. The relative gantry test evaluates the accuracy of gantry rotation, using MV images of the MPC phantom at eight gantry angles (45° apart), not compared with the nominal gantry angle. The TrueBeam MPC reports the maximum offset between the angle determined, using the MV imaging system and the nominal gantry angle.^10^ The threshold for both absolute and relative gantry positioning on the TrueBeam is ±0.3°. The Halcyon MPC relative gantry angle test is slightly different to that on the TrueBeam: the images taken at 0° and 270° are used as the reference for the absolute angle. The threshold for both absolute and relative gantry positioning on the Halcyon is ±0.5°.

#### 2.D.2 Error detection tests

The MPC test of the absolute gantry position was evaluated for Halcyon linac. We intentionally miscalibrated the gantry position by 0.3°, 0.5°, 1.0°, and 2.0°, all in the same direction, with the actual rotation measured using a digital level. The values reported by MPC were compared to these expected values.

The MPC test for the relative gantry rotation was assessed by removing the phantom mounting bracket and then mounting the MPC phantom on a stepper motor controlled rotational stage (Velmex Inc.) [Fig. 2(d)]. This allowed us to rotate the phantom by 0.1° to 5° degrees at different points between acquisitions of the eight gantry angle images acquired during the automated MPC sequence. The accuracy of the rotational stage was verified, using a digital level. The reported MPC result was compared with the known offsets introduced by the phantom rotations.

## 3 RESULTS

The results of the error detection tests are summarized in Table 2.

**Table 2:** Results of comparison between known offset and MPC check results

Test	Machine		Difference between known value and MPC			
			Average difference	Standard deviation	Minimum difference	Maximum difference
% Output^a^	Halcyon		1.31	0.48	0.70	1.95
	TrueBeam		0.51	0.28	0.16	0.84
Absolute gantry angle (degree)	Halcyon		0.01	0.01	0.00	0.03
Couch translation (mm)	Halcyon	Longitudinal	0.02	0.03	0.00	0.06
		Lateral	0.09	0.06	0.04	0.15
		Vertical	0.02	0.02	0.01	0.05
	TrueBeam	Longitudinal	0.02	0.02	0.01	0.05
		Lateral	0.10	0.09	0.01	0.22
Virtual to actual isocenter couch translation (mm)	Halcyon	Longitudinal	0.18	0.06	0.13	0.27
		Lateral	0.28	0.15	0.06	0.43
		Vertical	0.33	0.02	0.31	0.36
MLC position (mm)	Halcyon		0.05	0.10	−0.01	0.23

a Output data is reported for intentional reductions in output by up to 5%. The differences between MPC and Profiler reported output increased with larger changes in output.

To evaluate the reproducibility of the MPC tests, the process was repeated three times, and the range in the reported values calculated. The ranges were 0.44% and 0.15% for the reported changes in output and uniformity, respectively, 0.04° for gantry measurements, and 0.05 mm for couch shifts in any direction.

### 3.A Beam output and uniformity

**Figure 3:**
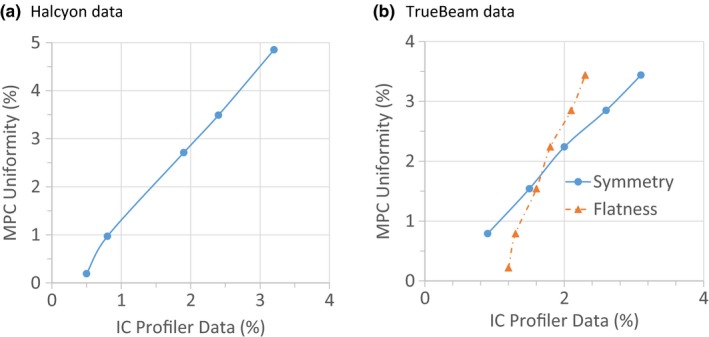
(a) Halcyon data: Uniformity measured using the MPC compared with symmetry measured, using the IC profiler. (b) TrueBeam data: Uniformity measured using the MPC compared with symmetry and flatness measured, using the IC Profiler.

We compared the change in beam output reported by the MPC and the output change measured by the IC Profiler. For both the TrueBeam and Halcyon linacs, the MPC tended to overreport the change in linac output compared with the IC Profiler. This difference increased with larger changes in beam output, and was <2% for clinically realistic changes of <5%. This overreporting results in a conservative QA results, and is consistent with use for daily QA. (it is also probably related to beam hardening as we added additional solid water — this is discussed below). Changes in the Halcyon symmetry were measured by the IC profiler, and plotted against the uniformity measured by the Halcyon and TrueBeam MPC (Fig. 3). For TrueBeam, we also included flatness. Note, the flattened 6‐MV beam is used in the TrueBeam MPC tests in this work.

### 3.B MLC position

The differences between the MLC offset measured by MPC and the intentionally miscalibrated values are listed in Table 2. The average difference was 0.05 mm and the maximum difference was 0.23 mm, which we observed when we miscalibrated the MLC position by 5.25 mm. Standard deviations, minimum and maximum differences are given in Table 2.

### 3.C Couch translation

In order to assess any impact of the translational stages on the experimental setup, MPC was performed with the phantom attached to the stage, but without any induced shifts. The results were within the ranges found when MPC was repeated multiple times (reported above), indicating that the presence of the translational stage itself does not impact the MPC results.

The difference between the phantom offset shifted by the linear translation motor and the MPC measurement for both TrueBeam and Halcyon linacs is shown in Table 2. For the TrueBeam, the average difference between known shifts and MPC measurements was 0.01 mm for both longitudinal and lateral directions. For the Halcyon, the average differences between the known shifts and values reported by the MPC couch shift tests were 0.02 longitudinally, 0.07 laterally, and 0.01 mm vertically. In addition, the average differences between the known shifts and values reported by the MPC virtual to isocenter couch translation tests were 0.18 longitudinally, 0.26 laterally, and 0.03 mm vertically. Standard deviations, minimum and maximum differences are given in Table 2.

### 3.D Gantry rotation

The differences between the absolute gantry angle measured by the Halcyon MPC and the known miscalibrated values are also listed in Table 2. The average difference was 0.014°. The maximum difference was 0.03°, which happened when we miscalibrated the gantry position by 2°. Standard deviations, minimum, and maximum differences are given in Table 2.

The detailed measurement results of the MPC relative gantry rotation tests for both TrueBeam and Halcyon linacs are listed in Table 3. The difference between the rotating angles by the motor and the MPC results was maximum when we rotated the phantom at four out of eight gantry positions during imaging. The repeatability of these measurements was evaluated by repeating the measurement where the phantom was intentionally rotated by 1° for one position, 5 times. The MPC reported 0.82 ± 0.03°.

**Table 3:** Detailed results of MPC relative gantry rotation tests

Machine	Angle offset (degree)	Number of gantry positions where phantom was rotated						
		1	2	3	4	5	6	7
Halcyon		MPC results (degree)						
	0.1	0.05	0.02	0.04	0.05	0.04	−0.04	
	0.3	0.19	0.16	0.16	0.13	−0.14	−0.16	
	0.5	0.37	0.31	0.28	0.23	−0.26	−0.31	
	1	0.8175	0.69	0.58	0.47	−0.57	−0.69	
	1.5	1.21	1.04	0.89	0.71	−1.19	−1.09	
	2	1.64	1.42	1.19	0.96	−1.19	−1.42	
	5	4.29	3.68	3.09	2.47	−3.07	−3.65	
TrueBeam	0.5	0.51	0.48	0.41	0.35	−0.4	−0.46	−0.48
	1	0.99	0.88	0.8	0.66	−0.79	−0.88	−0.94
	2	2.16	1.89	1.58	1.28	−1.57	−1.88	−2.12

## 4 DISCUSSION

Our testing has shown that the Machine Performance Check, as deployed with the Halcyon and TrueBeam treatment device, is capable of detecting changes/errors in beam constancy and mechanical parameters to a level that is appropriate for use for daily QA.

### 4.A Beam output and uniformity

These results indicate that the MPC can detect changes in beam output and uniformity with sufficient accuracy/precision for daily QA. One important weakness in our study is in the way in which we changed the beam output. We inserted solid water into the beam, which has the additional impact of hardening the radiation beam — thus, the changes detected by the MPC test are likely and also partially due to the overresponse of the portal imager to the low energy component of the beam, resulting in larger differences than may be found without this effect. Barnes and Greer tested the ability of the MPC to accurately detect output by intentionally adjusting the linac output (TrueBeam) and found that MPC output agreed with ion chamber to within 0.17%,^5^ which is better agreement than found in our study. Clivio also found better agreement between MPC and ion chamber.^4^

Our approach was to try to change the output (and uniformity, and other parameters) using external means. Thus, when both results are considered together (internal and internal adjustments), this adds confidence to our conclusion that the MPC can detect changes in beam output and uniformity. Furthermore, although we did not report on the day‐to‐day stability of these measurements, other authors^4^, ^5^ have reported that the MPC beam output measurements accurately tracked other independent measurements of beam output and flatness and symmetry over an extended period of time. Therefore, the MPC appears to provide an adequate measurement of the beam output and quality for daily QA purposes. Although the MPC may also be appropriate for monthly beam constancy checks, we do not believe that this has been proven with a sufficient degree of confidence, so monthly QA with independent equipment is still warranted.

### 4.B MLC position

The accuracy of the MPC MLC position test was well within the 1–2 mm tolerance suggested by TG142 for daily checks of the collimator size indicator (All beam collimation on the Halcyon is performed, using the MLCs), indicating that the MPC is sufficient for daily QA. The MPC does not explicitly include some of the more detailed MLC tests suggested for less frequent checks (weekly, monthly or annual). It should be reasonably simple for the vendor to add some of these tests to a future version of the MPC. It should be noted that the MLC version available in our preclinical Halcyon is an early version, where the upper MLC bank is slaved to the lower bank. Additional tests will be necessary when the vendor releases a version where the upper and lower banks operate independently.

### 4.C Mechanical checks (Couch translation and gantry rotation)

The largest disagreement between the couch translation (or absolute position) and the intentionally inserted error was less than 0.5 mm, well within the 1 to 2 mm tolerances suggested in TG‐179 for couch shift accuracy (monthly and daily tolerances, respectively). These results support the use of the MPC for checking these parameters. Although we do not yet have any data on the expected failure modes of the gantry rotation (e.g., is it even possible for the system to be incorrect for a small arc?), given that the tolerance for gantry angle indicators suggested by the AAPM TG 142 is 1.0°, the results presented here indicate that (with the current vendor threshold), the use of the MPC is appropriate for testing gantry angle errors. These results, therefore, support the use of the MPC checks to replace some of the mechanical checks that are often performed on a daily basis. They may also replace some of the mechanical checks that are performed monthly.

There are some limitations of our study. We did not perform any tests of the coincidence of imaging and treatment isocenters. Our early version of the Halcyon did not include kilovoltage imaging, so this will have to be tested once released. Our version of the MLC was also a preclinical version (including the lower bank being slaved to the upper bank, as mentioned above). Our MLC tests were also limited to changing the calibration for a single MLC leaf, although this limitation is somewhat mitigated because earlier authors have investigated this issue (Barnes). Finally, we did not assess changes in the MPC over time. We do not expect this to be a concern for mechanical tests, and other authors have followed the MPC output results over relatively extended periods.

In summary, our tests have shown that the MPC can detect errors in the beam constancy and geometric parameters with an accuracy that is appropriate for use as daily QA. In many cases, the MPC also has the potential to replace monthly QA checks, but additional work (especially regarding constancy) is needed before a solid conclusion can be given regarding the use of MPC for monthly QA.

## 5 CONCLUSION

We showed that the MPC of both Halcyon and TrueBeam linacs can detect errors in the beam constancy and geometric parameters with an accuracy that means the MPC is appropriate for use as daily QA.
